# Development and Characterization of Bio-Based Composite Films for Food Packing Applications Using Boiled Rice Water and *Pistacia vera* Shells

**DOI:** 10.3390/polym15163456

**Published:** 2023-08-18

**Authors:** Vinnarasi A. Raj, Karthikumar Sankar, Pandiarajan Narayanasamy, Innasi Ganesh Moorthy, Natesan Sivakumar, Shyam Kumar Rajaram, Ponmurugan Karuppiah, Mohammed Rafi Shaik, Abdulrahman Alwarthan, Tae Hwan Oh, Baji Shaik

**Affiliations:** 1 Department of Biotechnology, Kamaraj College of Engineering and Technology, K. Vellakulam, Virudhunagar 625701, Tamil Nadu, India; vinstarlion@gmail.com (V.A.R.); skarthikumar@gmail.com (K.S.); 2 Department of Mechanical Engineering, Kamaraj College of Engineering and Technology, K. Vellakulam, Virudhunagar 625701, Tamil Nadu, India; narayananx5@gmail.com; 3 School of Chemical Engineering, Vellore Institute of Technology, Vellore 632014, Tamil Nadu, India; igmoorthy@yahoo.co.in; 4 Department of Molecular Microbiology, School of Life Sciences, Madurai Kamaraj University, Madurai 625021, Tamil Nadu, India; microshivaak@gmail.com; 5 Department of Botany and Microbiology, College of Science, King Saud University, P.O. Box 2455, Riyadh 11451, Saudi Arabia; pkaruppiah@ksu.edu.sa; 6 Department of Chemistry, College of Science, King Saud University, P.O. Box 2455, Riyadh 11451, Saudi Arabia; mrshaik@ksu.edu.sa (M.R.S.); awarthan@ksu.edu.sa (A.A.); 7 School of Chemical Engineering, Yeungnam University, Gyeongsan 38541, Republic of Korea; taehwanoh@ynu.ac.kr

**Keywords:** *Pistacia vera*, biocomposite film, biodegradable, packaging properties

## Abstract

Customer demand for natural packaging materials in the food industry has increased. Biocomposite films developed using boiled rice water could be an eco-friendly and cost-effective packaging product in the future. This study reports the development of bio-based films using waste materials, such as boiled rice water (matrix) and *Pistacia vera* shells (reinforcement material), using an adapted solution casting method. Several film combinations were developed using various concentrations of plasticizing agent (sorbitol), thickening agent (oil and agar), and stabilizing agents (Arabic gum, corn starch, and *Pistacia vera* shell powder). Various packaging properties of the film were analyzed and examined to select the best bio-based film for food packaging applications. The film fabricated with *Pistacia vera* shell powder in the biocomposite film exhibited a reduced water solubility, swelling index, and moisture content, as compared to polyethene packaging material, whereas the biocomposite film exhibited poor antimicrobial properties, high vapor transmission rate, and high biodegradability rate. The packaging properties and characterization of the film indicated that the boiled rice water film with *Pistacia vera* shell powder was suitable for packaging material applications.

## 1. Introduction

The demand for packaging materials in the food sector has increased, and worldwide attention has been focused on natural biopolymers for the development and improvement of food packaging materials. The abundant use of synthetic polymers in the food packaging industry has resulted in harmful effects on humans and the environment. In the last two decades, enormous quantities of toxic plastic packaging chemicals in the form of microplastics have been dumped into marine environments, polluting the marine ecosystem. Groh et al. reported that various plastic packaging chemicals exhibit toxic and bioaccumulating properties [1]. Alabi et al., stated that the food packaging sector releases large amounts of toxic chemicals into the marine system in the form of microplastics [2]. Moreover, synthetic plastic materials can become adulterants during high-temperature food processing. Currently, a large quantity of non-degradable plastic materials is discarded, which has initiated several studies to develop novel eco-packaging substances that can be considered sustainable raw materials [3].

Different biopolymers have been selected from various biological sources, such as plants, polysaccharides, and microbial biomass, to develop eco-friendly films. The packaging ability of the materials and their quality parameters were analyzed using the physicochemical, thermal, and strength properties of the films [4]. Therefore, food researchers have adopted different methodologies to overcome the use of synthetic films by developing various starch-and modified starch-based films for food packaging [5]. Bioactive packages were developed using starch from several rice varieties, such as black, red, and white rice, and the film properties were analyzed based on color, opacity, thickness, and water resistance. Changes in the type and concentration of starch and plasticizing agents result in films with different packaging properties [6]. Numerous biopolymers have been developed as alternative sources of synthetic films because of their biodegradability and eco-friendly nature. However, the use of biopolymers leads to poor mechanical and barrier properties. Marichelvam et al. reported that rice-starch- and corn-associated biomaterials can act as biocomposites for various applications [7]. Their study also reported that the tensile strength (TS) of the film improved when a combination of different biopolymers was used with rice starch.

Numerous biopolymers have been developed as alternative sources of synthetic films because of their biodegradability and eco-friendly nature. However, the use of biopolymers leads to poor mechanical and barrier properties. To overcome this problem, various biopolymers have been added as fillers and nanocomposites [8]. Additionally, the use of natural biopolymers creates problems during film development owing to their brittleness and fragility. To solve this problem, various biodegradable plasticizers have been incorporated into biopolymer materials [9]. However, Prasetyo et al. [10] reported that the concentration of the plasticizers used in the film influences the physicochemical parameters of the film; therefore, an appropriate amount of the ingredient should be used to develop a good film. Furthermore, an optimum biofiller concentration should be used in the film to reinforce and improve its mechanical properties [11]. To decrease the brittleness of corn starch (CS) films and to improve their water vapor permeability, different types of plant-derived oil were incorporated into the film [12]. The water resistance of a film prepared using mung bean starch was improved by incorporating hydrophobic substances, such as sunflower seed oil, into the film. The addition of oil reduced the water solubility and strength of the film [13]. Wittaya concluded that several physical and chemical treatment combinations should be used to improve the properties of film-forming rice starch [14]. Appropriate treatment should be selected to improve the mechanical characteristics of rice using product-based films.

Bandyopadhyay et al. [15] reported that rice starch and chitosan blends exhibited superior film properties after ultrasound treatment. Similarly, Jayaraj et al. [16] reported that the water resistance of films containing rice starch was enhanced by replacing cellulose with nanocellulose. The nanocellulose–rice-starch blend exhibited improved film-forming properties. Therefore, this study focused on boiled rice water (a by-product of the cooking of rice) to prepare cost-effective thin polymeric films for food packaging applications. Furthermore, the packaging properties and characteristics of the developed films were studied. Rice starch and flour were used as raw materials for film preparation. Research on cooked rice water-based film preparation is limited. Therefore, this research may facilitate the preparation of cost-effective composite films with enhanced properties that are applicable to the food industry for packaging purposes.

## 2. Materials and Methods

### 2.1. Preparation of Raw Materials

Waste boiled rice water was collected from hostels and stored in sterile conical flasks. The other ingredients used to expand the biocomposite film were agar (AR), sorbitol, cornstarch, coconut oil, Arabic gum (AG), and *Pistacia vera* shells. AR was purchased from AK Seaweed, Ramanathapuram, Tamil Nadu. *Pistacia vera* shells were collected from waste and pulverized to a particle size < 45 μm. Sorbitol was purchased from Sigma-Aldrich. CS and coconut oil were purchased from a shop and used as thickening agents. Several film combinations were developed using various concentrations of the ingredients, such as a plasticizing agent (sorbitol), thickening agent (oil), AR, and fillers, such as AG, CS, *Pistacia vera* shell powder (PVS), and both PVS and CS (CSPVS).

### 2.2. Development of Biocomposite Film

The biocomposite films were prepared according to the following protocol. One milliliter of sorbitol was blended with 100 mL boiled rice water, and the solution was magnetically stirred for 30 min. The ingredients of the films were varied to develop five different types of films. The compositions and concentrations of the ingredients in the films are presented in Table 1. The solution was then gelatinized using microwave radiation. The microwave-irradiated solution was homogenized and subjected to ultrasonication to improve the integration of starch with biopolymers. A casting method was used to develop the resulting thin-film solution.

### 2.3. Determination of Packaging Properties of the Biocomposite Film

The quality and integrity of the physio-chemical and food packaging properties of the biocomposite film were evaluated. Biological properties, such as the antimicrobial activity, microbial barrier properties, and biodegradability, were analyzed.

#### 2.3.1. Thickness and Density

The film-holding capacity of the biocomposite films was indirectly estimated by analyzing its thickness and density. The thicknesses of 2 × 2 cm areas of the biocomposite films were measured at different points using a screw gauge. The densities (g cm^−3^) of the biocomposite films were determined according to the protocol described by Salgado et al. [17].

#### 2.3.2. Water Solubility

A modified Wang et al. protocol was used to determine the solubility of the film in water [18]. The initial weight of the film (2 cm × 2 cm) was calculated after the film was dried in a hot air oven at 105 °C for 3 h. The dried films were then immersed in 15 mL distilled water for 24 h at room temperature and agitated at 100 rpm. The final weight of the films (2 × 2 cm) was calculated after drying the water-soaked films again for 3 h. The dried samples were weighed again. The solubility of the samples was determined using Equation (1).

(1) $$Water\;Solubility\left(\%\right)=\frac{Initial\;weight\;of\;sample-Final\;weight\;of\;sample}{Initial\;weight\;of\;sample}\times 100$$

#### 2.3.3. Moisture Content

The moisture content of the bio-based films was evaluated according to the procedure described in [19]. The initial weight of the biocomposite films (2 × 2 cm) was determined by drying the film at 100 °C in an oven for 3 h. The dried samples were weighed, and the final weights of the films were determined.

(2) $$Moisture\;content\left(\%\right)=\frac{Initial\;weight\;of\;sample-Final\;weight\;of\;sample}{Initial\;weight\;of\;sample}\times 100$$

#### 2.3.4. Swelling Index

Samples (2 × 2 cm) were dried at 100 ± 1 °C for 3 h and weighed (A). The oven-dried samples were immersed in 50 mL distilled water at room temperature for 5 min. The soaked film was wiped with a filter paper. The swollen samples were weighed (B). The swelling index of the samples was calculated using Equation (2).

(3) Swelling Index%=(A−B)A×100

where A and B are the weights of the absorbed and dried samples, respectively.

#### 2.3.5. Vapor Transmission Rate

A container containing calcium chloride was closed with a sample of the bio-based film firmly fixed on top [20]. The container was then placed in a desiccator with potassium nitrate at 37 °C [21]. The films were weighed daily using an analytical balance for 5 d, and the vapor transmission rate was calculated using Equation (4) [22,23,24].

(4) $$WVTR=\frac{w\times x}{t\times A}\;$$

where WVTR is the water vapor transmission rate, x is the average thickness of the film, A represents the permeation area, and x/t is calculated by linear regression from the points of weight gain and time during a constant-rate period.

#### 2.3.6. Determination of the pH of the Film-Forming Solution

The pH of the film-forming solution was determined at room temperature using a digital pH meter with a glass electrode after standardization with 4 and 7 pH buffers.

#### 2.3.7. Optical Properties

A UV spectrophotometer (UV 1601 Spectrophotometer; Shimadzu, Kyoto, Japan) was used to determine the film transparency. The film samples were cut into rectangular pieces and placed on the inner side of the spectrophotometer cell. The transmittance of the films was determined at 600 nm [25].

#### 2.3.8. Biodegradability Tests

Biodegradation experiments were performed as described in [26]. The specimens (5 cm × 5 cm) were buried 3 cm beneath the soil surface and maintained at room temperature. The specimens were periodically weighed using an analytical balance until they were completely degraded in the soil. The biodegradability of the films was indirectly determined by calculating their weight reduction.

#### 2.3.9. Microbial Barrier Properties

Sterilized biocomposite films (5 cm × 5 cm) were aseptically placed on the opening of a test tube containing sterile nutrient broth, and the corners were sealed with Parafilm. The control test tube was left open. All test tubes were incubated at room temperature. After the third day, the spread plate technique was used to determine microbial growth [27].

#### 2.3.10. Antimicrobial Properties

The antimicrobial activity of the biocomposite films against *Escherichia coli* (*E. coli*) and *Bacillus subtilis* (*B. subtilis*) were evaluated using AR disk diffusion. *E. coli* and *B. subtilis* were cultured in a nutrient medium at 37 °C in a shaking incubator for 24 h. The antibacterial activity was evaluated using the procedure described in [28].

#### 2.3.11. Mechanical Properties

The mechanical properties of the prepared biofilms, such as the TS and elongation at break, were measured using a 0.1 N load cell at a crosshead speed of 30 mm/min. The average TS values were calculated and expressed as the mean ± standard deviation.

### 2.4. Characterization of the Biocomposite Films

The functional characteristics of the starch-based films were studied using Fourier transform infrared (FTIR) spectroscopy, and the molecular interactions between starch and other biopolymers were studied. Scanning electron microscopy (SEM) and X-ray diffraction (XRD) were performed to analyze the structure of the films, which helps to understand the surface properties of the newly developed starch films. Chemical analyses of the biocomposite films were performed using FTIR to analyze the functional chemical groups present in the film. The film samples were prepared using the press method. The samples were crushed and mixed with KBr pellets, and the spectra were recorded at room temperature [29]. FTIR was performed in the spectral range of 750–4000 cm^−1^. XRD analyses were performed using an advanced diffractometer system with Cu Kα radiation with a wavelength of 1.54 Å. The spectra of the film samples were recorded at a generator voltage and current of 40 kV and 20 mA, respectively [30]. The crystallinity and amorphous % of the films were calculated using Bragg’s law and Scherrer’s equation, respectively [31]. Surface imaging of the biocomposite film was performed using SEM (Center for Nanoscience and Nanotechnology, The Gandhigram, Rural Institute-Deemed University, Dindugal, Tamil Nadu, India). SEM was used to image the surface of the film. The electron beam, which results in a high-resolution image of the film, is the basis for SEM analysis. SEM images of the biocomposite film were acquired with a resolution of approximately 5–10 nm. The images were acquired at an accelerating potential of 10 kV. To visualize the morphology and surface behavior of the starch granules, high-resolution SEM was performed [32]. Thermogravimetric analyses were performed at 30–500 °C using a simultaneous thermal analyzer to measure the thermal characteristics of the biocomposite films in a nitrogen environment at a step-up heating rate of 10 °C/min. TGA/DTA analyses were performed on all the films. The weights of the sample films were recorded before analysis.

## 3. Results and Discussion

### 3.1. FTIR Analysis

**Figure 1:**
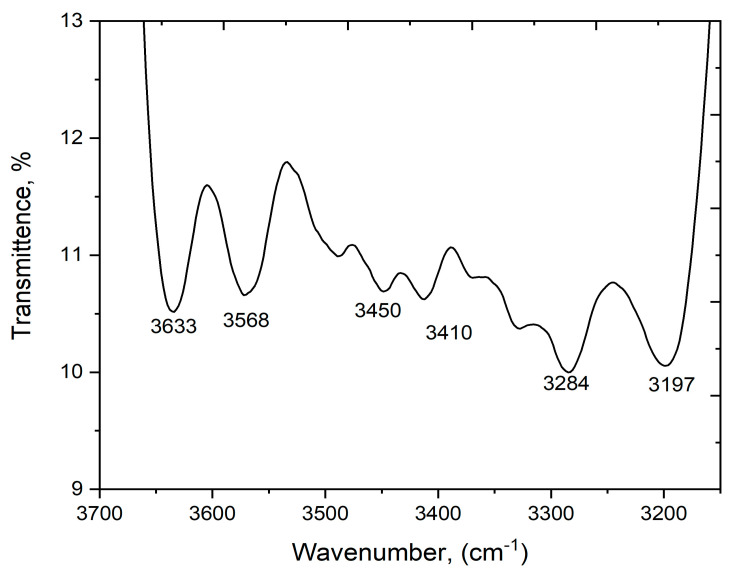
Fourier transform infrared (FTIR) spectrum of boiled rice water.

Figure 1 shows the FTIR analysis results of the boiled rice water. The absorbance peak at 3633.89 cm^−1^ corresponds to the O–H stretching present in water molecules. The peaks at 2920–2928 cm^−1^ correspond to methylene linkages in starch molecules, whereas those at 3100–3700 cm^−1^ indicate alcohol bonds [32]. The FTIR spectrum of CSPVS exhibited a reduction of the broad bands at 3500–3100 cm^−1^, which represents the O–H bonds. The major peak at 2926.01 cm^−1^ indicates the presence of C–H groups. The sharp peak at 1458 cm^−1^ indicates the attachment of starch molecules to water [33]. Similarly, the C–O group at 1413.82 cm^−1^ indicates the presence of C–O bonds in the native rice boiled water.

**Figure 2:**
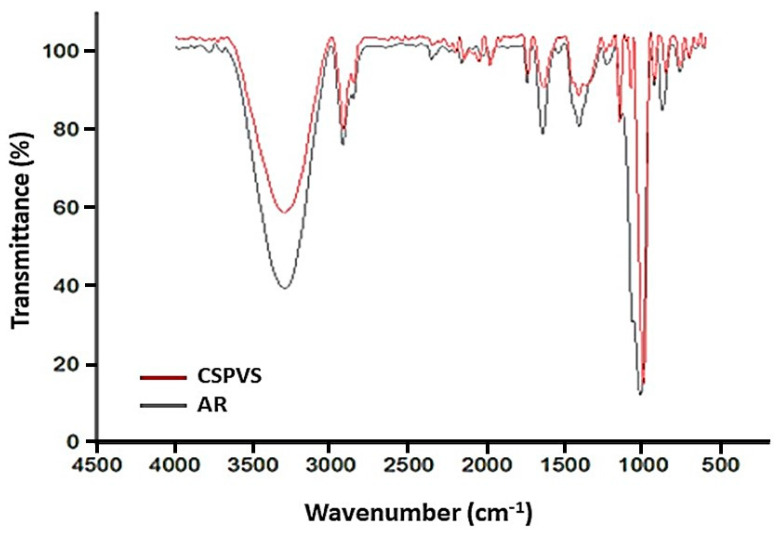
FTIR spectra of the agar (AR) and corn starch and *Pistacia vera* shell powder (CSPVS) films.

A comparative analysis of the FTIR spectra of the AG and CSPVS films was performed to identify structural modifications in the films during the study period. The FTIR bands at 950–1050, 1018, and 1047 cm^−1^ indicate the amorphous character and crystalline order of the starch [33]. Similarly, the FTIR results in this study also showed a peak at 950–1050, indicating that the boiled rice water-based film was amorphous. Remarkably, the presence of new bands in the FTIR spectra of the AR/PVS blend films at 1151 and 600 cm^−1^ indicated an increase in the amylose content and presence of functional groups of lignin and hemicellulose. The sharp bands at 920–999 cm^−1^ were associated with α-(1–4) glycosidic linkage (C–O–C) and skeletal mode vibrations in rice starches mixed with PVS. The FTIR spectrum of the film exhibited wavenumbers ranging from 4000 to 500 cm^−1^ (Figure 2). The presence of –OH and –CH groups in the starch resulted in a peak at 3407 cm^−1^ [34]. The aromatic vibration of the benzene ring in lignin was observed at 1621 cm^−1^. The peak at 1621–1425 cm^−1^ corresponded to the C–O stretching in lignin. The broad peas that appeared at 1042 cm^−1^ was attributed to the –CO–C– pyranose ring skeletal vibration corresponding to the O–H stretching and bending vibrations of polysaccharide in cellulose [35].

### 3.2. SEM Analysis

**Figure 3:**
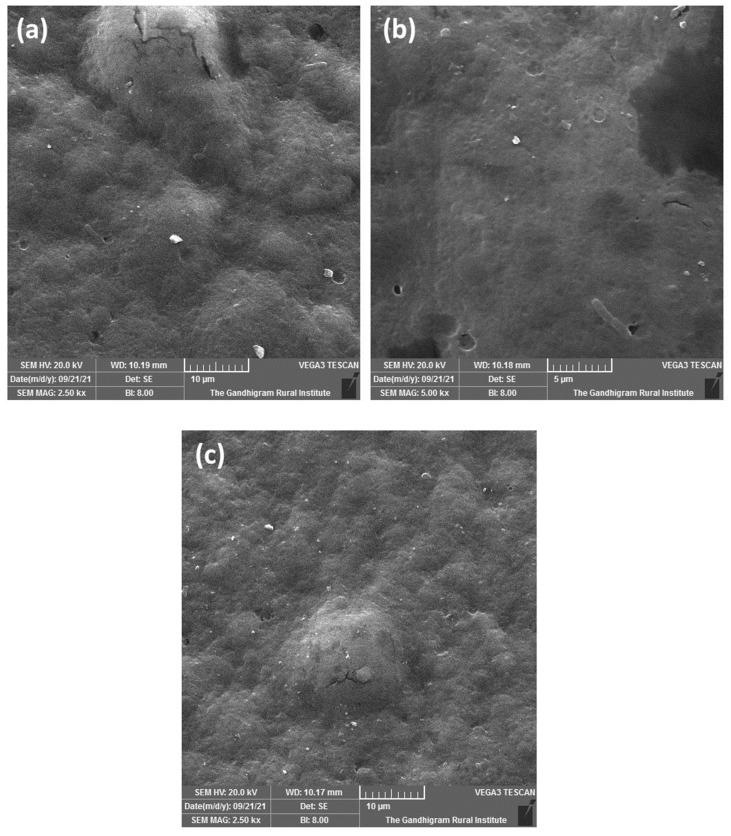
Scanning electron microscopy (SEM) images of the films fabricated using (**a**) AR, (**b**) corn starch (CS), and (**c**) CSPVS.

SEM was performed to study the structural morphology at the micron and submicron levels. Figure 3 shows the structural changes in the films with and without *Pistacia vera* shells. *Pistacia vera* shells are composed of hemicellulose and lignin. Figure 3a shows a film fabricated using AR, and Figure 3b shows a film fabricated using CS. Ahmad et al. [33] reported that after ultrasonication, starch nanoparticles were irregular in size and shape, similar to the results shown in Figure 3a. Furthermore, the film was rough and exhibited valleys owing to the non-uniform distribution of starch in the AR matrix. Staroszczyk and Janas [36] reported that subjecting starch to microwave radiation reduces its semi-crystalline nature to an amorphous structure. The microwave-treated sample in Figure 3b exhibited a smooth surface with few peaks. Therefore, it is necessary to improve the surface behavior of both the boiled rice water and *Pistacia vera* shells.

### 3.3. XRD Analysis

**Figure 4:**
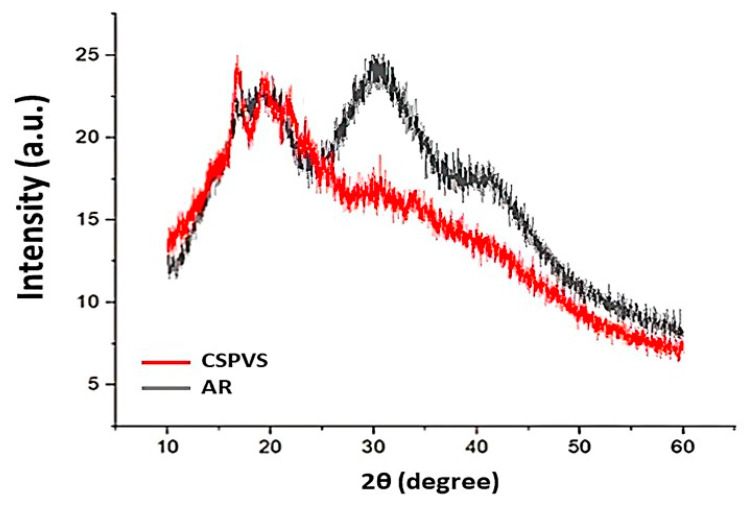
X-ray diffraction (XRD) analysis of the films fabricated using AR and CSPVS.

Movva and Kommineni [37] suggested that the degree of crystallinity is a key property for characterizing a material. Therefore, the crystallinity of the *Pistacia vera* shell-added film was determined and compared with that of the film without *Pistacia vera* shells, as shown in Figure 4. The rice starch films exhibited an amorphous structure after gelatinization. The crystallinity % and amorphous % of the biocomposite film fabricated using the *Pistacia vera* shells were 16.7 and 83.3%, respectively, and those of the films fabricated without the *Pistacia vera* shells were 19.3 and 80.7%, respectively. The addition of PVS to the starch-based film increased the quantity of amorphous peaks. The increase in the amorphous nature was attributed to molecular interactions between these biomaterials and the boiled rice water components.

### 3.4. Mechanical Properties

**Figure 5:**
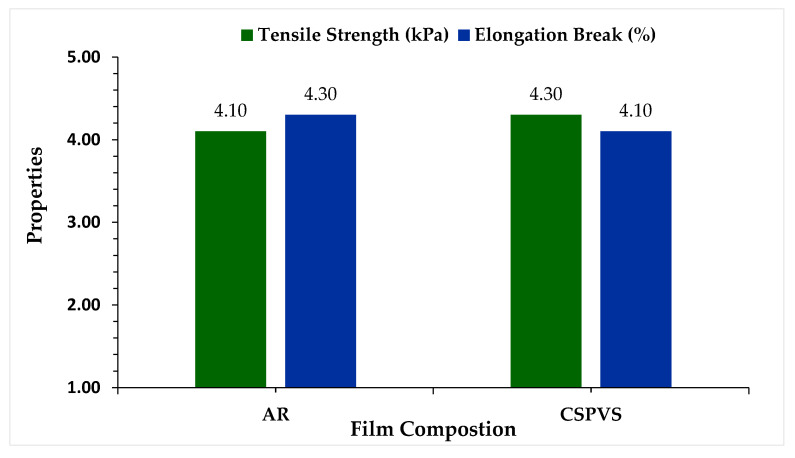
TS and elongation at break of the films fabricated using AR and CSPVS.

The maximum tensile stress sustained by the film under a particular tension test was considered as the TS. The TS and elongation at break are mechanical properties that can be used to evaluate any type of biocomposite film proposed for food packaging (Figure 5). The mechanical properties of a film are indirectly related to the maintenance and storage of food at a particular temperature. 

The composition of the film influences its stability and strength. Fathiraja et al. [38] reported that the TS of a film is influenced by the presence of AR with a plasticizer and stabilizing agent (Figure S1, see in Supplementary Materials). The elevated effect of AR on the TS has also been reported for AR-based edible films [39]. The TS value range of CSPVS films was higher than that of the AR films. Elongation at break is another important property for films used in the food packaging industry; however, the AR films exhibited better elongation-at-break values than the CSPVS films. This variation may be due to the composition of biopolymers and fillers added along with the boiled rice water. 

### 3.5. Thermal Properties 

**Figure 6:**
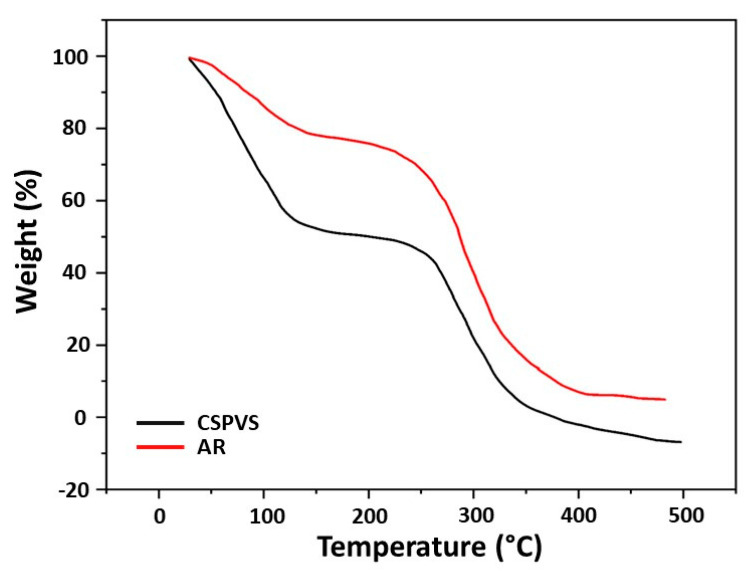
TGA analysis results of the films fabricated using AR and CSPVS.

Thermal analyses are used to study the stability of biomaterials at elevated temperatures, and the effect of heating on starch for the development of biocomposite films can be assessed. The TGA revealed three phases of biomaterial decomposition. The first phase (up to 110 °C) was attributed to the evaporation of water (Figure 6). The next stage occurred at 100–220 °C and accounted for 30.89% of the loss in weight, which was attributed to the dehydration and desorption of water associated with starch. These results show that gradual degradation occurred up to 300 °C. Above this temperature, the thermal stability gradually decreased and the biopolymers decomposed. The third phase (approximately 300–360 °C) accounted for approximately 33.95% of the weight loss. Indran et al. [40] reported that the maximum decomposition peak at 356 °C is attributed to the decomposition of cellulose and lignin. The temperature at which 60% decomposition occurs in Figure 6, approximately 269 °C, indicated that the addition of the *Pistacia vera* shells did not influence the thermal stability of the materials. Similar to the result of Wijaya et al. [41], the TGA curve exhibited a steep decrease at 320 °C, indicating that the principal decomposition of starch occurred at this temperature.

### 3.6. Thickness and Density

**Figure 7:**
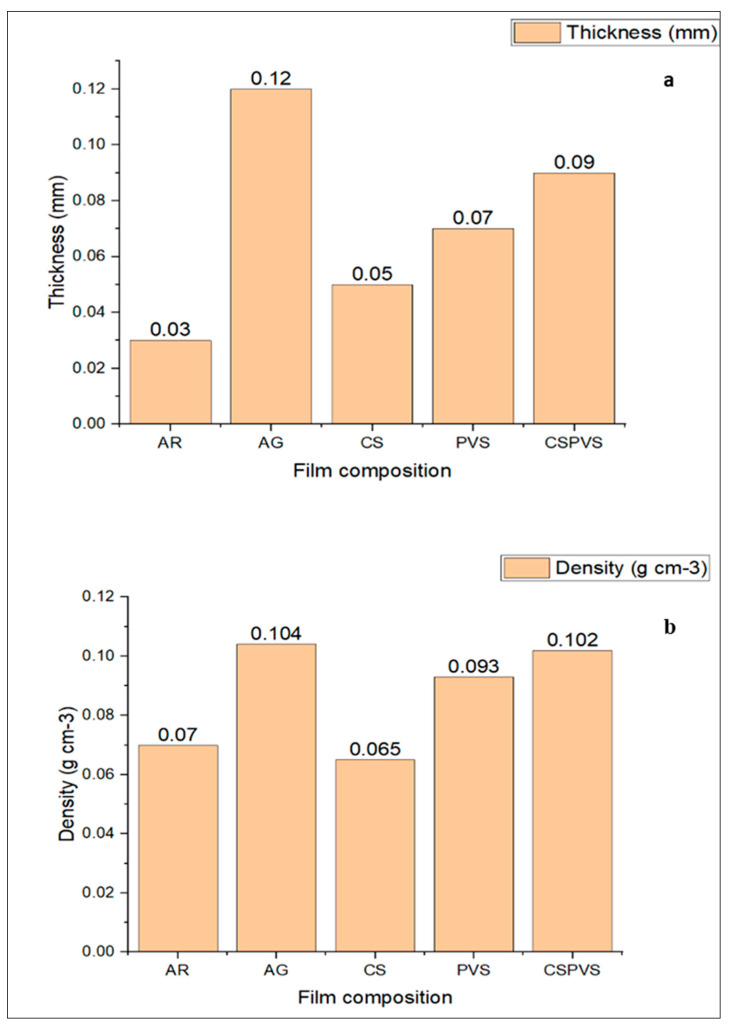
(**a**) Thickness and (**b**) density of different biocomposite films.

Bio-based packaging materials require toughness to bear the holding pressure. These strength parameters can be assessed using the physicotensile properties of the bio-based materials, such as thickness and density. The results (Figure 7) show that the thickness of all the biocomposite films progressively increased owing to an increase in the concentration of the biofiller added to the film. CS/*Pistacia vera* shell-based films had higher thickness values than the AR/CS-based films. These results may be linked to the large particle size of the *Pistacia vera* shells used as the filler. It has been reported that the bio-based film thickness can increase owing to the larger particle size of the added materials [6]. A direct proportional relationship between the thickness and density of the film was observed. The thickness of the film increased with an increasing film density.

### 3.7. Water Solubility

**Figure 8:**
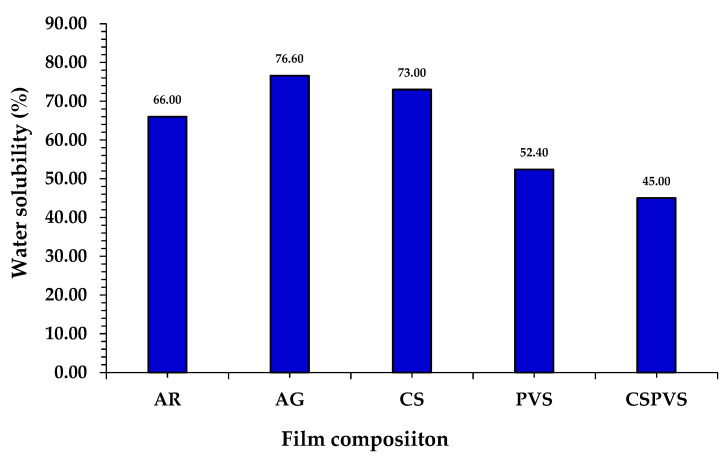
Water solubility of the different biocomposite films.

Biocomposite films that are susceptible to water content may not be suitable as packaging materials for stable food and meat. A good bio-based film is expected to maintain optimum humidity conditions for a period of time to prevent microbial growth on the film; therefore, determination of the water solubility of the starch-based film is very important [19]. Jumaidin et al. [42] suggested that the high water solubility of the film was due to the presence of high quantities of water-soluble and hydrophilic polysaccharides, such as carrageenan, in the seaweed used. However, when a hydrophilic compound hybrid with the fiber material was used to form a film, the film exhibited a reduced water solubility. Similar to this study, the film fabricated using AR exhibited a higher solubility in water than the film fabricated using AR and other biofillers. The solubility results in Figure 8 shows that the bio-based film produced using CVPVS exhibited a good performance, as compared with the films fabricated without CSPVS. For example, the film fabricated using AR exhibited 66% solubility, whereas the film fabricated using AR and AG exhibited a maximum solubility of 76.6%. Additionally, an increase in the PVS in the CS film decreased the water solubility of the film with a solubility of 45%. This variation may be due to the interaction between the water content and the biopolymers present in the biocomposite film.

### 3.8. Moisture Content

**Figure 9:**
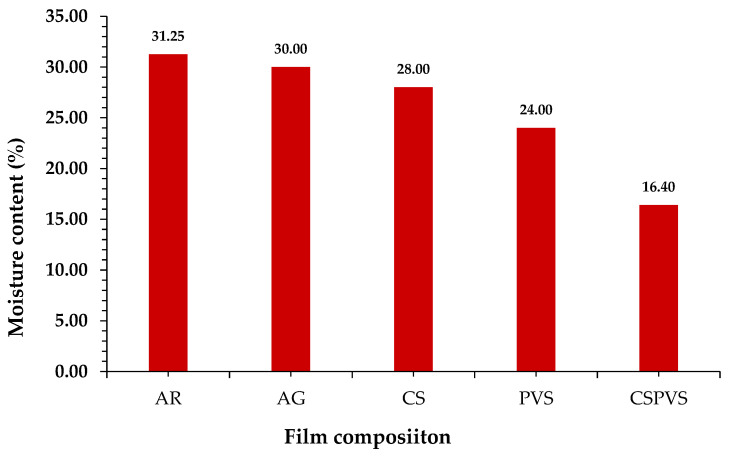
Moisture content of different biocomposite film.

Oluwasina et al. [19] suggested that the presence of moisture in food packaging systems can lead to food contamination. Therefore, a good packaging material is expected to not increase the moisture content of packaged substances. The moisture content results (Figure 9) shows that the CSPVS film had a lower moisture content (16.4%) than the AR film (31.25%). The moisture content varied according to the surface composition of the film. The film prepared with AR had a higher moisture content. Phan et al. [43] reported that the moisture content of AR-based films was higher than that of starch-based films. The variation in the moisture content of the biocomposite film was due to the hygroscopic and hydrophilic nature of the AR and other polysaccharides added along with the boiled rice water.

### 3.9. Swelling Index

**Figure 10:**
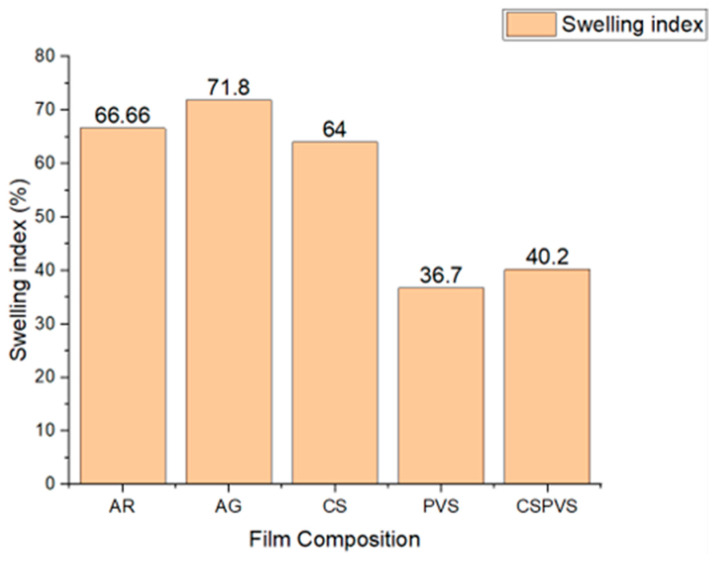
Swelling index of the different biocomposite films.

The swelling mechanism of hydrogels is based on the presence of hydrophilic functional groups in the biopolymers [44]. Abdollahi et al. [45] reported that a carboxymethyl cellulose film incorporated with an essential oil exhibited a reduced swelling index. This indicates that the affinity of water molecules for other biopolymers and the oil in the biocomposite film plays a major role in the swelling index of the film. Changes in the affinity of the water molecules indirectly influence the absorption of water by biocomposite films. As shown in Figure 10, the swelling index of the films fabricated using AR and AG were higher owing to the hygroscopic nature of AR. However, the swelling index of the film fabricated using *Pistacia vera* shells, as compared to the film fabricated using AR alone, was lower. The amylase content influences the swelling capacity and solubility of the film in water by changing the negatively charged elements within the rice starch granules [46].

### 3.10. Vapor Transmission Rate

**Figure 11:**
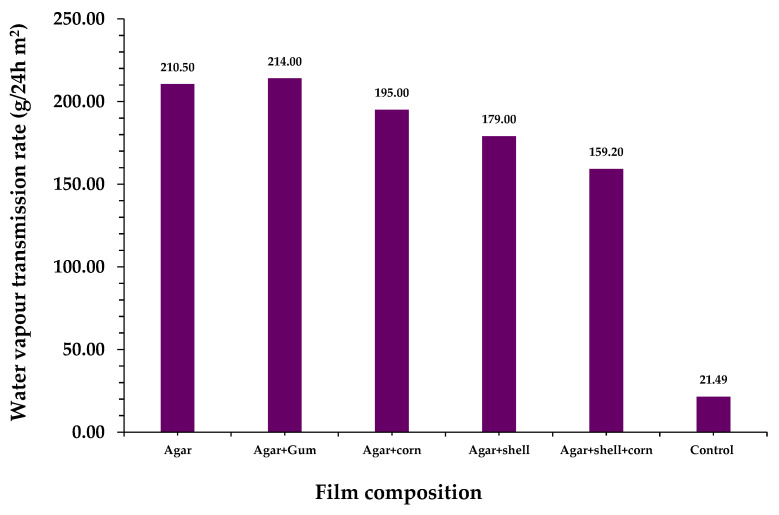
Vapor transmission rate of the different biocomposite films.

One role of food packaging films is to prevent the entry and transfer of water molecules from the environment to food samples. Therefore, the water vapor permeability should be low (Figure 11) [47]. The rate at which water vapor enters a starch film depends on the diffusivity and permeability of the film. The water molecular absorption ability and surface porosity of a film influences the vapor transmission rate of the food packaging system [48]. The hydrophilic characterization of starch films results in poor moisture resistance [49]. Based on the above study, the boiled rice water-based film also exhibited a lower vapor transmission rate than the control (polyethene sheet film). The film fabricated using PVS exhibited a lower vapor transmission rate (159.2 g/24 h m^2^), as compared with the film fabricated using AR (210.5 g/24 h m^2^). These variations may be due to the permeability of the water vapor entering the film.

### 3.11. Determination of pH under Casting Process Conditions

pH is an important factor that must be considered and determined to maintain the integrity and nature of the film. The acidity and alkalinity of the films were indirectly determined by the change in the hydrogen ion activity of the casting solution during film development. Sakkara et al. [50] reported that films prepared under different pH conditions exhibited variations in the physicochemical and mechanical properties, and stated that films fabricated at an alkaline pH were water repellent and flexible. Starch granules can change their shape and radius of gyration under different pH conditions, which may be due to degradation and hydration [51]. In this study, the pH of the casting film was determined during each film development process and did not change. They found that the initial pH was 6.5 during heating; oxidation of starch caused a slightly acidic pH condition. After homogenization, microwave treatment, and ultrasonication, the pH of the film casting solution was closer to pH 8. Similar to that reported by Sakkara [50], the film-forming properties of a biocomposite film fabricated using an alkaline solution are suitable for the packaging industry.

### 3.12. Determination of the Optical Properties of the Biocomposite Films

**Figure 12:**
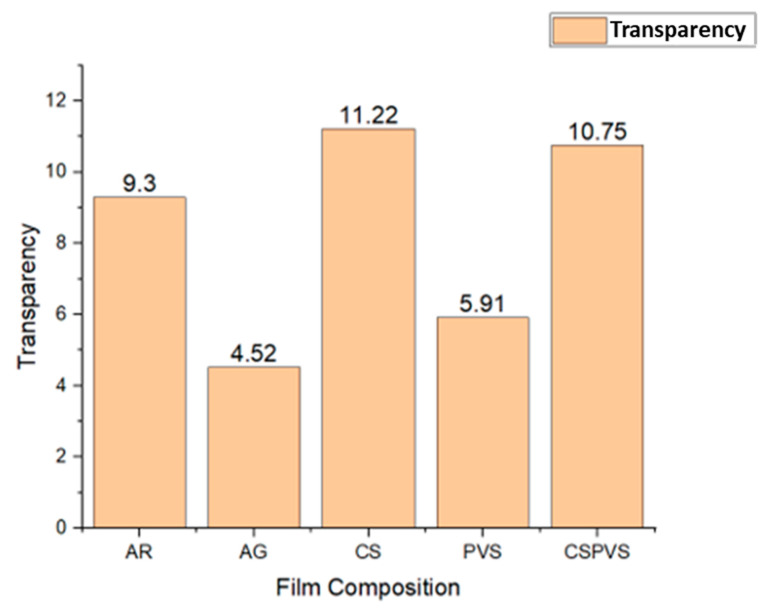
Transparency of the different biocomposite films.

In some food applications, the transparency of film packaging materials is an important parameter. The addition of PVS to the boiled rice water films decreased their transparency (Figure 12). The transparency of the CSPVS film was higher than that of the films in which greater quantities of PVS and AG were incorporated into the starch film. The decrease in transparency may be due to the light scattering of the *Pistacia vera* shell and AG from the PVS and AG films, respectively.

### 3.13. Biodegradability Test

**Figure 13:**
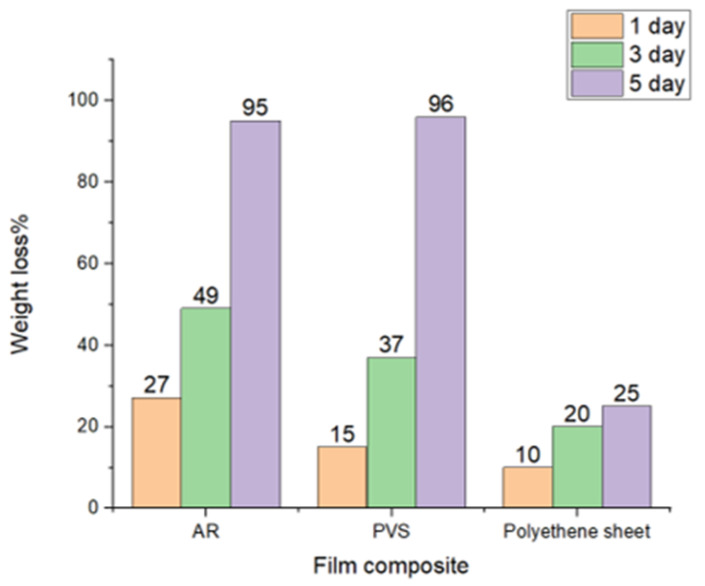
Biodegradability rate of the different biocomposite films.

Biodegradable packaging has recently become an alternative to chemically derived plastic [52]. Additionally, the structure and properties of biodegradable materials change due to environmental conditions, exposure to air, soil microorganisms, and UV radiation. A major portion of the degradation of biocomposite films is performed by tiny biotic organisms present in soil. These organisms determine the degradation rate because they contain enzymes that allow them to degrade film wastes [53]. The weight loss during degradation of the starch films is shown in Figure 13. The weight of the starch films decreased, leading to a total weight loss of >90% after 5 d. The CSPVS-based films exhibited the highest percentage of weight loss (96%), whereas the control (polyethene sheet) film exhibited the lowest percentage of weight loss (25%). This difference was attributed to the glycosidic bond linkages in starch and nondegradable chemical bonds in the plastic packaging film.

### 3.14. Microbial Barrier Properties 

The entry of microorganisms into food packaging materials causes food to decay. Good food packaging materials have strong microbial barrier properties; therefore, they can indirectly extend the shelf life of food and protect it from environmental microorganisms [54]. After 3 d of incubation, high turbidity was observed in the bottles covered with AR-based films. However, lower turbidity was observed in the bottles covered with the CSPVS film. This was also validated by the growth of microorganisms on nutrient AR plates for samples from the control bottles and by the lower microbial count from samples of the CSPVS film. The permeability of the bacterial spores to environmental conditions varied based on the porosity of the starch film. To improve the microbial barrier properties, antimicrobial agents should be blended with the starch material.

### 3.15. Antimicrobial Tests

Antimicrobial packaging is a new technology in which different active compounds are used in food packaging materials to extend the shelf life of packed food. Films can be used as an active packing material by preventing the entry of food-prone pathogens. This can be achieved by incorporating antimicrobial agents, such as essential oils, into the films [37,55]. A previous study reported that *Pistacia vera* shell extracts were active against Gram-positive bacteria, with bactericidal effects against *Listeria monocytogenes* (ATCC strains and food isolates), *Staphylococcus aureus*, and MRSA clinical isolates. However, in our study, the films fabricated using CSPVS exhibited no antimicrobial activity. Plates covered with films not containing PVS exhibited no inhibitory action. These results may be due to an overpopulation of selected cultures or an insufficient quantity of film for antimicrobial activity. Pyla et al. [56] reported that starch-based films impregnated with fresh tannic acid (FTA/starch film) and thermally processed tannic acid (PTA/starch film) were assessed for the inhibition of *E. coli* O157:H7 and *Listeria monocytogenes*. Disc-diffusion assay revealed that the PTA/starch film exhibited a larger clear zone around the film on the bacterial lawn than the FTA/starch film at the same tannic acid concentrations (0.45–4.5 mg/disc). Therefore, a natural antimicrobial agent must be mixed with the film to obtain better results.

## 4. Conclusions

This study highlights the potential of developing an eco-friendly and cost-effective biocomposite film for food packaging applications using waste boiled rice water and PVS. The films were fabricated by incorporating various concentrations of sorbitol as a plasticizing agent; oil and AR as thickening agents; and AG, CS, and PVS as stabilizing/filler agents. The CSPVS biocomposite film exhibited several positive packaging properties, including reduced water solubility (45.00%), swelling index (40.20%), and moisture content (16.40%), as compared with traditional polyethylene packaging materials. These properties indicate an enhanced resistance to water and moisture, which can contribute to improved food preservation and shelf life. However, the biocomposite film showed limitations in terms of its antimicrobial properties and vapor transmission rate (159.20), as compared with conventional packaging materials. Additionally, although the biodegradability rate (96.00%) was relatively high, further improvements in this aspect may be desirable to fully meet ecofriendly standards. Considering the overall packaging properties and film characterization, the biocomposite film fabricated using boiled rice water and PVS has emerged as a suitable and promising packaging material. The use of this material has the potential to contribute significantly to reducing the environmental impact and addressing the increasing customer demand for natural and sustainable packaging solutions in the food industry. Further research and optimization efforts could lead to better biocomposite films, address their limitations, and make them even more attractive for widespread food packaging applications.

## Figures and Tables

**Table 1:** Composition of the biocomposite films.

S. No.	Composition of Film	Quantity
1.	Boiled rice water	100.00 mL
2.	Agar	1.00 g
3.	Corn starch	300.00 mg
4.	*Pistacia* shell powder	150.00 mg
5.	Sorbitol	1.00 mL
6.	Oil	100.00 μL

## Data Availability

Data contained within the Article.

## Supplementary Materials

The following supporting information can be downloaded at: https://www.mdpi.com/article/10.3390/polym15163456/s1, Figure S1. Diverse bio-composite film from different stabilizing agents; (a) Agar + corn, (b) Alginate + corn, (c) Alginate + Gum, (d) Agar + Gum, (e) Agar + Pistacia shell, (f) Alginate + Pistacia shell, (g) and Agar + Pistacia shell shell + corn.
