# Supplementary material for: Development and Characterization of Bio-Based Composite Films for Food Packing Applications Using Boiled Rice Water and Pistacia vera Shells
Source: Polymers (Basel). 2023 Aug 18;15(16):3456. doi: 10.3390/polym15163456 (PMC10457870; doi:10.3390/polym15163456)
Supplement: Supplementary file 1 [file polymers-15-03456-s001.zip › polymers-2500599-supplementary.pdf]

polymers

Supplementary Materials

Development and Characterization of Bio-Based Composite Films for Food Packing Applications Using Boiled Rice Water and *Pistacia vera* Shells

Vinnarasi A. Raj [1], Karthikumar Sankar [1], Narayanasamy P. [2], Ganesh Moorthy I. [3], Sivakumar N. [4], Shyam Kumar Rajaram [1,*], Ponmurugan Karuppiah [5], Mohammed Rafi Shaik [6], Abdulrahman Alwarthan [6], Tae Hwan Oh [7] and Baji Shaik [7,*]

[1] Department of Biotechnology, Kamaraj College of Engineering and Technology, K. Vellakulam, Virudhunagar 625701, Tamil Nadu, India; vinstarlion@gmail.com (V.A.R.); skarthikumar@gmail.com (K.S.)
[2] Department of Mechanical Engineering, Kamaraj College of Engineering and Technology, K. Vellakulam, Virudhunagar 625701, Tamil Nadu, India; narayananx5@gmail.com
[3] School of Chemical Engineering, Vellore Institute of Technology, Vellore 632014, Tamil Nadu, India; igmoorthy@yahoo.co.in
[4] Department of Molecular Microbiology, School of Life Sciences, Madurai Kamaraj University, Madurai 625021, Tamil Nadu, India; microshivaak@gmail.com
[5] Department of Botany and Microbiology, College of Science, King Saud University, P.O. Box 2455, Riyadh 11451, Saudi Arabia; pkaruppiah@ksu.edu.sa
[6] Department of Chemistry, College of Science, King Saud University, P.O. Box 2455, Riyadh 11451, Saudi Arabia; mrshaik@ksu.edu.sa (M.R.S.); awarthan@ksu.edu.sa (A.A.)
[7] School of Chemical Engineering, Yeungnam University, Gyeongsan 38541, Republic of Korea; taehwanoh@ynu.ac.kr
* Correspondence: kingshyam2003@gmail.com (S.K.R.); shaikbaji@yu.ac.kr (B.S.)

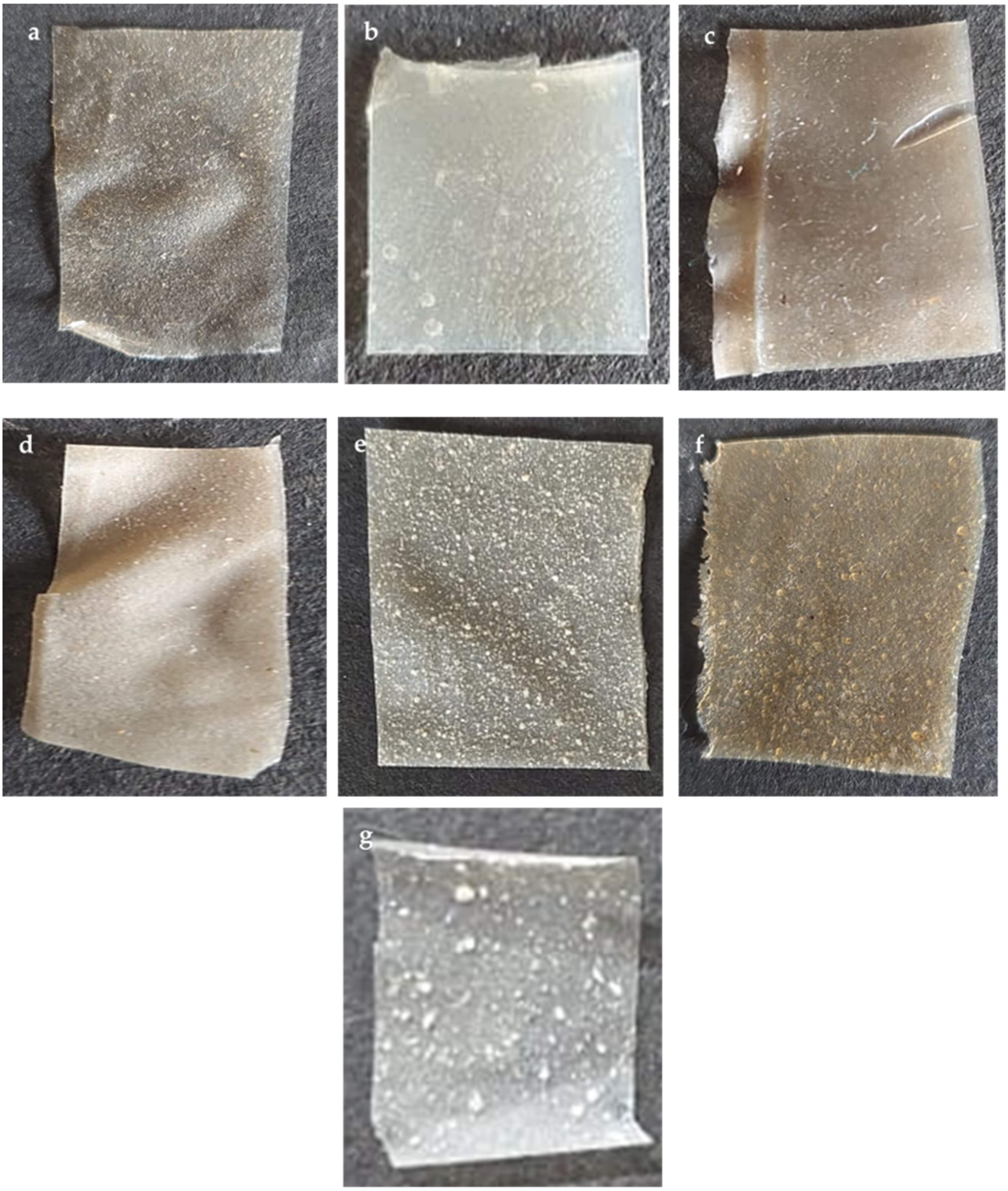

Figure S1. Diverse bio-composite film from different stabilizing agents; a) Agar+corn, b) Alginate+corn, c) Alginate+Gum, d) Agar+Gum, e) Agar+ Pistacia shell, f) Alginate+ Pistacia shell, g) Agar+ Pistacia shell shell+corn.
